# Carboxymethyl β-Cyclodextrin Assistance for the 4-Nitrophenol Reduction Using Cobalt-Based Layered Double Hydroxides

**DOI:** 10.3390/ijms25126390

**Published:** 2024-06-09

**Authors:** Alexia Demeester, Fatima Douma, Renaud Cousin, Stéphane Siffert, Gwladys Pourceau, Anne Wadouachi, Anne Ponchel, Eric Monflier, Sébastien Noël

**Affiliations:** 1 Unité de Catalyse et Chimie du Solide (UCCS), UMR 8181, Université de Lille, CNRS, Centrale Lille, Université d’Artois, rue Jean Souvraz, SP 18, 62300 Lens, France; demeesteralexia@gmail.com (A.D.); fatimadouma@yahoo.fr (F.D.); anne.ponchel@univ-artois.fr (A.P.); eric.monflier@univ-artois.fr (E.M.); 2 Unité de Chimie Environnementale et Interactions sur le Vivant (UCEIV, UR 4492), Université du Littoral Côte d’Opale, 59140 Dunkerque, France; renaud.cousin@univ-littoral.fr (R.C.); stephane.siffert@univ-littoral.fr (S.S.); 3 Laboratoire de Glycochimie et des Agroressources d’Amiens (LG2A) UR 7378, Institut de Chimie de Picardie, Université de Picardie Jules Verne, 33 rue St Leu, 80039 Amiens, France; gwladys.pourceau@u-picardie.fr (G.P.); anne.wadouachi@u-picardie.fr (A.W.)

**Keywords:** layered double hydroxides, carboxymethyl β-cyclodextrin, cobalt, nitroaromatics

## Abstract

Cobalt-aluminum-layered double hydroxides containing carboxymethyl β-cyclodextrin (CMβCD) were synthesized by coprecipitation and evaluated as a cobalt source for the 4-nitrophenol reduction in an aqueous medium. Several physicochemical techniques (XRD, FTIR, TGA) indicated the intercalation of the anionic cyclodextrin without damages to the hydrotalcite-type structure. These lamellar cobalt-aluminum hybrid materials (CoAl_CMβCD) were evaluated in the 4-nitrophenol reduction and showed higher activities in comparison with the CMβCD-free standard material (CoAl_CO_3_). To rationalize these results, a set of experimental controls going from physical mixtures of CoAl_CO_3_ with different cyclodextrins to other cobalt-based materials were investigated, highlighting the beneficial effects of both the layered double hydroxide and CMβCD-based hybrid structures. CMβCD also showed a beneficial effect as an additive during the 4-nitrophenol reduction. CoAl_CO_3_, dispersed in a fresh CMβCD solution could be re-used for five successive cycles without the loss of activity.

## 1. Introduction

According to the US Environmental Protection Agency, 4-nitrophenol (4-NPhOH) has been listed as one of the 129 organic chemicals that are carcinogenic and harmful for humans, animals, and plants [1]. More generally, the elimination of toxic nitro-aromatic compounds, largely contained in effluents from industrial sources, has become an environmental priority for several decades. Among the approaches which are described in the literature such as microbial degradation [2], adsorption [3], and photocatalysis [4,5,6], the chemical reduction of 4-NPhOH is an interesting approach which is increasingly reported [7,8]. This reaction generally occurs in an aqueous medium in the presence of a reducing agent, such as sodium borohydride (NaBH_4_), or hydrazine (NH_2_NH_2_) in the presence of a metal source [9,10]. In order to reduce the cost of the chemical processes and to minimize their environmental impact, transition metal nanoparticles [11,12,13,14,15] or bulk transition metal oxides [16,17,18,19,20] including for instance Co_3_O_4_ [16,17,18] have also been reported.

Among transition metal-based materials, layered double hydroxides (LDH) instigate a growing interest in a wide range of applications, such as energy storage, drug delivery, and nanomedicine, but also pollutant remediation or catalysis [21]. LDH, also known as hydrotalcite-like compounds, can be written as follows: [M(II)_a_ M’(III)_b_ (OH)_2a+2b_] (A^n−^_b/n_) yH_2_O. They are built up by the periodic stacking of positively charged di- and trivalent metal hydroxides (M(II), M’(III)), octahedral layers, and negatively charged interlayer galleries consisting of anions (A^n−^) and water. As mentioned above, the use of LDH, as a bulk material or support for metal nanoparticles in catalysis, has increased in an exponential manner [22,23,24,25,26,27,28]. LDH-supported metal nanoparticles have also shown efficient activities towards the 4-NPhOH reduction [29]. For example, palladium nanoparticles, supported on NiFe-based LDH completely degraded 4-NPhOH in 5 min, with a turnover frequency value (the number of molecules converted per catalyst active site per time) of 597.66 h^−1^, which was about 17 times higher than that measured on a commercial Pd/C [30]. CoAl-based LDH-supported cobalt nanoparticles also showed high catalytic activities with a pseudo-first order rate constant of 86 (±3) min^−1^ at 25 °C for a catalyst dose of 1 g L^−1^ (as Co) and an initial 4-NPhOH concentration of 0.2 mM [31]. CoAl-based LDH as a noble metal free material could also be used for the reduction of nitro compounds, as recently reported by Nunes et al. in 2021 [32]. While gold nanoparticles immobilized on methionine-intercalated CoAl-based LDH exhibited high activities (0.129 min^−1^ for 2,6-dinitrophenol, 0.122 min^−1^ for *p*-nitrobenzylalcohol and 0.040 min^−1^ for *p*-nitroaniline), the parent LDH with chloride as an intercalating agent was found to follow it closely in terms of catalytic performance (0.078, 0.064 and 0.050 min^−1^ respectively). These results were promising and pave the way for using CoAl-based LDH materials as a cobalt source for reducing nitro compounds.

In this context, the idea was to investigate the 4-NPhOH reduction, using CoAl-based LDH as a metal source, in the presence of a supramolecular agent for concentrating the pollutant molecule close to the LDH material in water. Cyclodextrins (CDs) consisting of a macrocyclic ring of glucose subunits joined by α-1,4 glycosidic bonds, exhibit a truncated conical structure [33]. The cavity exhibits hydrophobic properties, allowing CDs to accommodate a large variety of hydrophobic molecules through the formation of a host-guest inclusion complex [34]. Moreover, the intercalation of CDs in LDH has already been studied, especially with CDs bearing carboxylate or sulfonate groups. For instance, some teams have examined the synthesis of LDH modified with cyclodextrins bearing carboxylate groups and applied them to the uptake of organic compounds [35,36]. To the best of our knowledge, no applications of chemical pollutant reduction using cyclodextrin-based hybrid LDH were reported.

In this present study, cobalt-aluminum-based LDH-containing carboxymethyl β-CD (CMβCD) were synthesized by a one-pot co-precipitation approach, characterized through different physicochemical techniques (XRD, TGA, FTIR, SEM-EDS) and tested as a cobalt source for the degradation of 4-NPhOH, in the presence of sodium borohydride. Several series of controls were carried out to emphasize the beneficial effect of the hybrid nature of the cobalt-based materials, but also the LDH structure on the reduction reaction efficiency. Complementary experiments were also performed to have a deeper understanding of the role played by the different partners during the 4-NPhOH reduction in an aqueous medium. Recycling experiments were finally investigated to evaluate the recovery of the hybrid materials.

## 2. Results

### 2.1. Characterization of Cobalt-Based LDH

The detailed synthetic procedure is given in the Experimental section. Briefly, the cobalt-aluminum LDH-based materials were synthesized by coprecipitating at pH 10.5 and 60 °C the respective nitrate salts, i.e., Co(NO_3_)_2_·6H_2_O and Al(NO_3_)_3_·9H_2_O, with or without the use of CMβCD (random distribution, 3.2 carboxymethyl groups on average per cyclodextrin unit) as the intercalating agent (see ESI–Figure S1). Two different initial concentrations of CMβCD in water were investigated (0.03 and 0.06 M). These levels of concentration were chosen in order to ensure the total substitution of the carbonate anions by CMβCD {with respect to the control LDH based on cobalt and aluminum and having the theoretical formula [Co_6_Al_2_(OH)_16_](CO_3_)·4H_2_O} [29]. After 24 h of aging, the resulting LDH materials were filtrated, washed with water, then finally dried overnight at 60 °C. The hybrid materials prepared from CMβCD are denoted as CoAl_CMβCD[X], where X refers to the initial CMβCD concentration used during the co-precipitation step, while the control CoAl-based LDH prepared without cyclodextrin assistance is denoted as CoAl_CO_3_.

Several physicochemical characterizations were conducted on the synthesized hybrid materials to confirm that the LDH phase was obtained, but also to determine the presence and quantity of CMβCD incorporated in the materials. 

**Figure 1:**
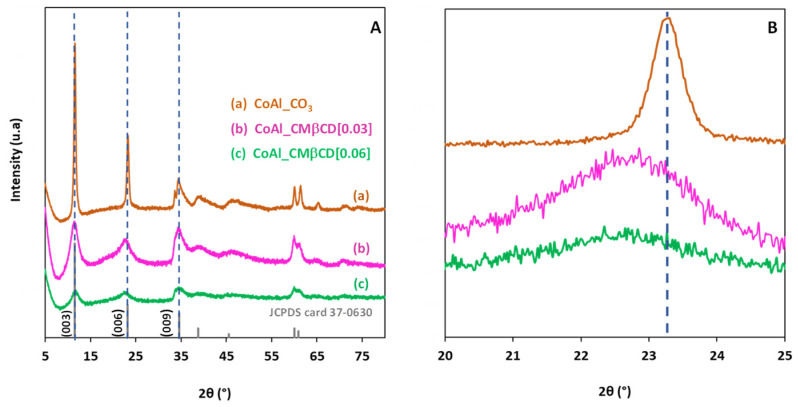
XRD patterns of the CoAl LDH materials prepared with and without CMβCD at (**A**) [5–80] 2θ range and (**B**) [20–25] 2θ range. The diffraction peaks of the JCPDS card number 37-0630 are included for comparison purposes.

XRD analysis was first performed to identify the crystal structure of the different materials, and the XRD patterns are shown in Figure 1A. The X-ray diffractogram of the control CoAl_CO_3_ (prepared without using CMβCD) exhibited a typical hydrotalcite-like structure (JCPDS n° 37-0630) containing Co^2+^ and Al^3+^ in the layers and carbonate anions intercalated in the LDH interlayer space, in agreement with the formula [Co_6_Al_2_(OH)_16_]CO_3_·4H_2_O. The presence of well-defined peaks at 2θ = 11.53°, 23.20°, and 34.60° attributed to the (003), (006), (009) reticular planes, respectively, revealed a good crystallinity of the LDH phase for the control CoAl_CO_3_. Based on the position of the diffraction plane (003), the calculated basal spacing was estimated at 0.76 nm, and this value was consistent with the presence of interlayer carbonates [37]. In the case of CoAl_CMβCD [0.03] and CoAl_CMβCD [0.06], the three characteristic diffraction peaks were still visible in the diffraction patterns, demonstrating that the synthesis protocol in-volving CMβCD effectively led to the formation of the LDH phase. However, the broadening of the peaks in XRD indicated that the size of the crystalline domains was clearly affected by the presence of the oligosaccharides during the synthesis. The higher the concentration of CMβCD, the wider the half width of the diffraction peak, and thus the smaller the average crystallite size (3.7 nm for CoAl_CMβCD [0.06] vs. 4.1 nm for CoAl_CMβCD [0.03] and 18.7 nm for CoAl_CO_3_). We observed also that the position of the XRD (006) peak slightly shifted toward lower 2θ angles with CMβCD (see Figure 1B), revealing that a certain lattice expansion occurs. This could be a first sign of the intercalation of the CMβCD anions in the interlayer of CoAl LDH [38].

**Figure 2:**
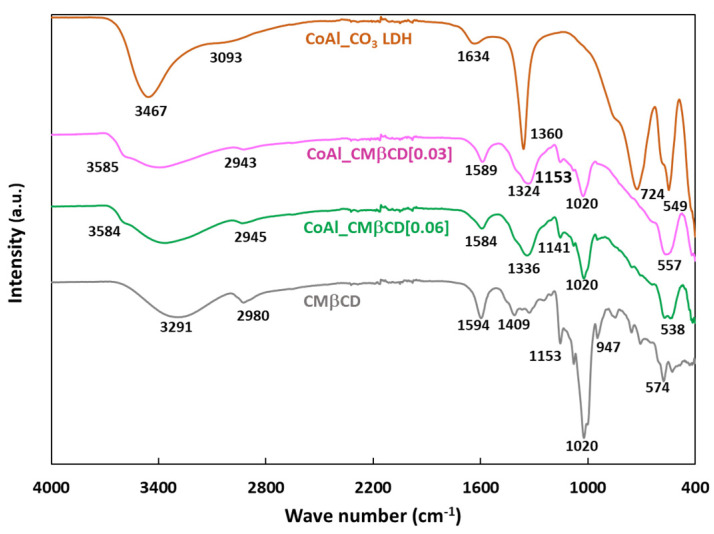
FTIR spectra of CoAl_CO_3_ and CoAl_CMβCD[X] with X = 0.03 and 0.06. For comparison, the FTIR spectrum of CMβCD was also included.

The CoAl LDH materials were further characterized using Fourier Transformed Infrared (FTIR) spectroscopy. Figure 2 plots the FTIR spectra of the parent CoAl_CO_3_ and those modified with CMβCD.

The first information given by the FTIR analysis was the confirmation that the LDH structure was formed for all materials, as previously demonstrated by XRD. Thus, the bands below 800 cm^−1^ could be assigned to metal-oxygen vibrations in the lattice of the LDH (724, 549 and 400 cm^−1^ for Co-O-Al, Co-O and Al-O respectively) [39]. On closer examination of the FTIR spectrum of CoAl_CO_3_, the band at 1360 cm^−1^ (antisymmetric carboxylate stretch vibration) is typically attributed to the presence of carbonates. In addition, the shoulder at about 3100 cm^−1^ corresponded to the stretching modes of the OH groups, which are linked to carbonates [40]. This spectrum also exhibited broad diffuse features between 3400 and 3500 cm^−1^ (υ (O-H)) and 1634 cm^−1^ (δ(H_2_O)) attributed to the intercalated water molecules [41,42]. For the CoAl_CMβCD[X] hybrid samples (with X = 0.03 and 0.06), we observed nearly identical features in the lattice spectral region below 800 cm^−1^, confirming that the LDH phase was indeed formed. It was also obvious that the vibration modes attributed to the carbonates appeared to be much lower than on the control, as though some of them may have been substituted with cyclodextrins. In fact, from 800 to 1200 cm^−1^, the FTIR spectra exhibited a set of bands typical of the fingerprint of cyclodextrin structure where the observed peaks can be associated to complex modes of the CH_2_OH groups, pyranose ring vibration and glycosidic C-O-C bonds. Notably, the band at 947 cm^−1^ could be specifically attributed to the skeletal mode of β-cyclodextrin molecules involving α-1,4 linkage [43,44]. Another indication of the presence of CMβCD moieties in the CoAl LDH structure was the presence of two additional peaks at ca. 1594 and 1409 cm^−1^ assigned to the antisymmetric and symmetric stretch vibrations of carboxylates (coming from CMβCD). The stretching bands of C-H (2750–2950 cm^−1^) were also detected by FTIR, showing the same type of spectral envelopes as that measured on bulk CMβCD. It should be noted that, for both hybrid materials, a shoulder was observed at high wavenumbers (typically 3585 cm^−1^), which was related to O-H stretching vibrations for free hydroxyl groups. These may be connected to a partial disruption of the water H-bonding network due to the intercalation of CMβCD, thus leading to more isolated hydroxyls [40]. Taken together, all of these results confirm the successful modification of the CoAl LDH phase by CMβCD without compromising the chemical integrity of the latter.

**Figure 3:**
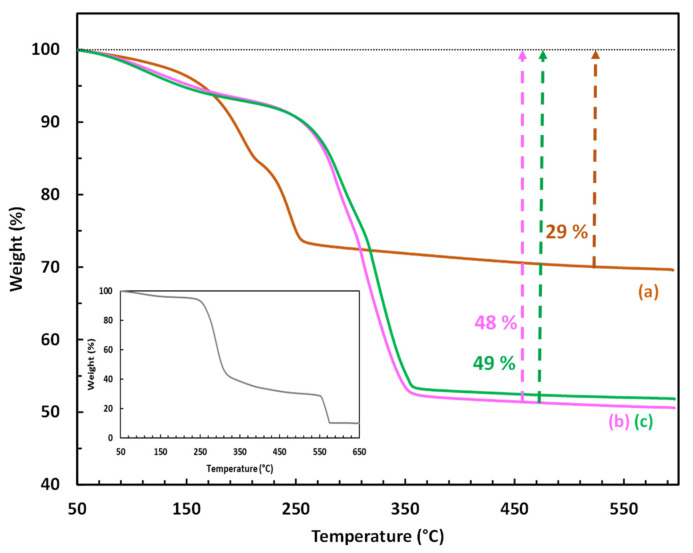
TGA profiles of (a) the LDH-based materials (CoAl_CO_3_; (b) CoAl_CMβCD [0.03]; (c) CoAl_CMβCD [0.06]) obtained under air flow. CMβCD profile has been given in the box.

To confirm the presence and determine the amount of CMβCD in the hybrid LDH, TGA was also employed (Figure 3). The thermal decomposition of the control LDH (CoAl_CO_3_) occurred in three consecutive steps: (i) the first weight loss (3%) between 50 and 150 °C corresponds to the removal of physisorbed water, (ii) the second weight loss (12%) between 150 and 215 °C suggests the elimination of the intercalated water molecules, and (iii) the third weight loss (14%) between 215 and 325 °C is associated with the decomposition/destruction of the LDH framework by a simultaneous process of dehydroxylation and decarbonation [45]. At 550 °C, the total weight loss achieved was 29%, leaving cobalt and aluminum oxides as solid residues. Regarding the samples CoAl_CMβCD [0.03] and [0.06], their thermograms followed a very similar profile, but shifted to significantly higher temperatures. The total weight losses were also found to be significantly higher (48 and 49%) compared to that measured on the parent CoAl LDH (29%). The presence of CMβCD is mainly responsible for these differences. Indeed, bulk cyclodextrins are known to decompose in air in a major step between 250 and 400 °C [46]. A careful examination of the TG profiles of the hybrid LDH indicated that the breakdown of the LDH structure (third step) appears to occur only after the cyclodextrins have undergone significant thermal degradation (282 °C for X = 0.03 and 305 °C for X = 0.06). The TG results indicated that the weight percent of CMβCD that could be incorporated into the two Co-Al LDH samples is relatively high, estimated to be ca. 28 and 24 wt%, corresponding to 0.21 and 0.19 mmol/g, respectively (see ESI-page S3 for the demonstration). This content appeared to relate to the maximum capacity for incorporation. Scanning electron microscopy (SEM) was carried out to observe the eventual morphological differences between the synthesized materials (see ESI–Figure S2), and the images were produced by using a backscattered electrons detector (BSE) in order to reveal also the elemental composition of the materials (Table 1). According to the scanning micrographs, no morphological differences have to be noticed between the CMβCD-free LDH and the hybrid materials.

For the control CoAl_CO_3_, the cobalt and aluminum contents, determined by EDS, were very close to the expected values, based on the corresponding LDH chemical structure [Co_6_Al_2_(OH)_16_]CO_3_·4H_2_O. For the hybrid materials, the cobalt content was lower and the carbon content much higher, which could be easily explained by the intercalation of CMβCD molecules inside the clay materials. Moreover, the value of 14% for the carbon element corresponds to the calculated value according to the thermogram with 28% of CMβCD. The presence of sodium was ascribed to the formation of ion pairs between the Na^+^ cation and the carboxylate groups of CMβCD during the material synthesis in an alkaline medium. All these physico-chemical analyses clearly showed the obtention of the LDH structure and the CMβCD incorporation after the coprecipitation of the metal cationic salts (Co^2+^ and Al^3+^) in presence of the carboxylate-functionalized βCD.

### 2.2. Reduction of 4-Nitrophenol in Aqueous Medium

**Figure 4:**
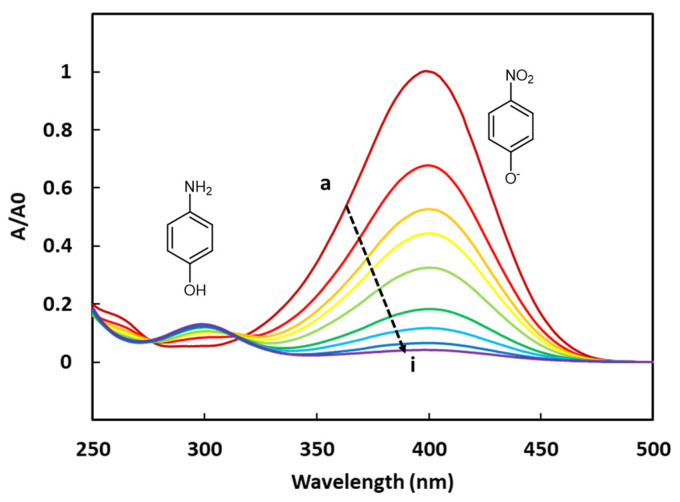
UV-vis spectra of the 4-NPhOH reduction in presence of CoAl_CMβCD [0.06] at different reaction times (a = 0, b = 2, c = 4, d = 7, e = 10, f = 15, g = 20, h = 25 and i = 30 min).

The 4-NPhOH reduction towards 4-aminophenol (4-APhOH) in an aqueous sodium borohydride solution (pH = 9.2) in the presence of the cobalt-based LDH, was monitored by UV-vis spectrophotometry. Before introducing the cobalt-based LDH, a color change (from pale to bright yellow) was observed, corresponding to the deprotonation of 4-NPhOH giving 4-nitrophenolate (4-NPhO^−^). This color change was not surprising, taking into account the pH value of the solution (9.2) and the pKa value of the (4-NPhOH/4-NPhO^−^) couple (7.2). The progress of the reaction was basically monitored by the disappearance of the 4-NPhO^−^ band at 400 nm (ε_4-NPhO_^−^ = 2.16 × 10^4^ L.cm^−1^.mol^−1^). The expected product (4-APhOH) band was centered at 300 nm (ε_4-APhOH_ = 4.15 × 10^3^ L.cm^−1^.mol^−1^) (Figure 4). It should be noticed that the absorbance values of the amino product are much lower than those of the nitro compound. This tendency has been clearly confirmed by preparing a synthetic equimolar mixture of 4-NPhOH and 4-APhOH in a sodium borohydride solution for allowing the total deprotonation of 4-NPhOH. The resulting solution was analyzed by UV-Vis spectroscopy, showing a much thinner absorbance for the 4-aminophenol compound (ESI—Figure S3).

**Figure 5:**
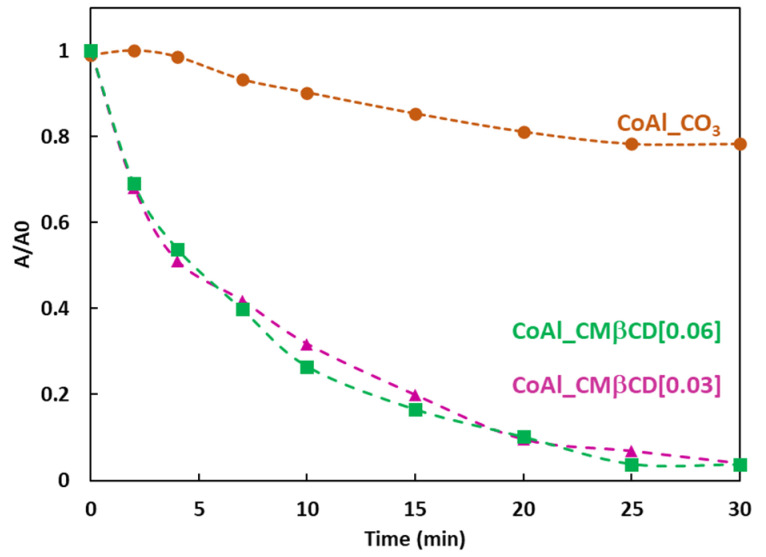
4-NPhOH reduction using NaBH_4_ in presence of LDH-based materials, followed by UV-Vis spectroscopy at 400 nm. Reaction conditions: 4-NP (2 µmol), NaBH_4_ (0.2 mmol), LDH-based material (20 mg), H_2_O (30 mL).

The performances of the CoAl_CMβCD[X] materials (X = 0.03 and 0.06 M) in the reduction of 4-NPhOH in a NaBH_4_ medium as a function of time are given in Figure 5. For comparison, the results obtained with the CMβCD free CoAl LDH control sample are given.

According to the absorbance measurements, the control CoAl_CO_3_ LDH showed a slow activity towards the reduction of 4-NPhOH. The decrease of the relative absorbance at 400 nm only reached 0.2 after 30 min of reaction. In the case of the two hybrid materials (CoAl_CMβCD[X] with X = 0.03 and 0.06 M), on the contrary, the complete disappearance of the 4-NPhO^−^ band was observed after 30 min of reaction. These differences in terms of activity are truly astonishing, given that the metal content of cobalt is similar for all materials (Table 1). This may be explained by the different chemical environments for the cobalt species which are more and less easily reducible by sodium borohydride. The similar activities for the two hybrids materials were not surprising, considering the same amounts of incorporated CMβCD units within the LDH structure through complementary TGA-EDS mapping analyses.

**Figure 6:**
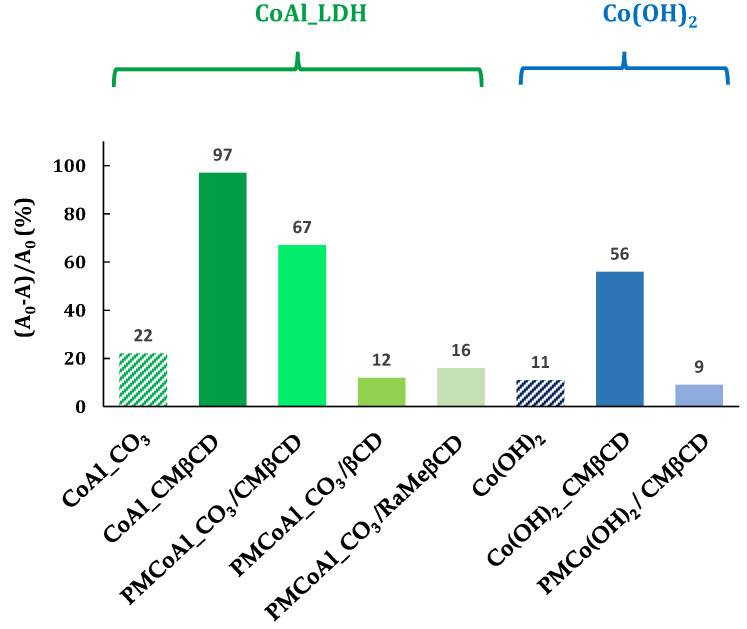
4-NPhOH reduction in a NaBH_4_ aqueous solution in the presence of cobalt-based materials. Reaction conditions: 4-NP (2 µmol), NaBH_4_ (0.2 mmol), cobalt-based material (20 mg), H_2_O (30 mL).

In order to understand the role of each part of these hybrid materials, the above-described results led us to carry out a series of control experiments (Figure 6).

The first series of controls was to know if the good activity was due to the hybrid structure, i.e., the CMβCD which was intercalated in the LDH structure. The 4-NPhOH reduction was performed using a mixture of CoAl_CO_3_ and the corresponding CMβCD amount (28 wt %), estimated by TGA analysis. This physical mixture (PMCoAl_CO_3_/CMβCD) has brought an activity improvement in comparison with the control CoAl_CO_3_. Undoubtedly, the activity of CoAl_CMβCD [0.06] is higher than the corresponding physical mixture. Other β-CDs, such as native and randomly methylated, have been evaluated (PMCoAl_CO_3_/βCD and PMCoAl_CO_3_/RaMeβCD, respectively). Contrary to CMβCD with 67% of conversion after 30 min, native β-cyclodextrin and randomly methylated β-cyclodextrin did not enhance the CoAl_CO_3_ activity (16 and 13% vs. 22%).

A second set of controls was carried out to extrapolate the beneficial effect of CMβCD to other cobalt-based materials towards the 4-NPhOH reduction. To respond to this request, two cobalt hydroxides were synthesized using the same procedure as LDH-based materials, with and without CMβCD with the same concentration as the hybrid LDH material, i.e., 0.06 mol·L^−1^ (ESI-page S4). The activity of these materials was then evaluated and compared to those of LDH-based cobalt compounds. The hybrid material (Co(OH)_2__CMβCD) showed a much higher activity than the cobalt (II) hydroxide without CMβCD, but at a lower manner than the LDH-based materials. Contrary to the physical mixture (CMβCD with CoAl_CO_3_), CMβCD brought no activity improvement in the presence of Co(OH)_2_.

In conclusion of all these results, the presence of CMβCD inside the materials (CoAl_CO_3_ or Co(OH)_2_) led to astonishing active materials towards the 4-NPhOH reduction. An activity improvement was also observed for the CoAl_CO_3_ solid in the presence of CMβCD, but not observed for other βCD derivatives (less than 20% after 30 min of reaction).

### 2.3. Cobalt-Based LDH Recycling

**Figure 7:**
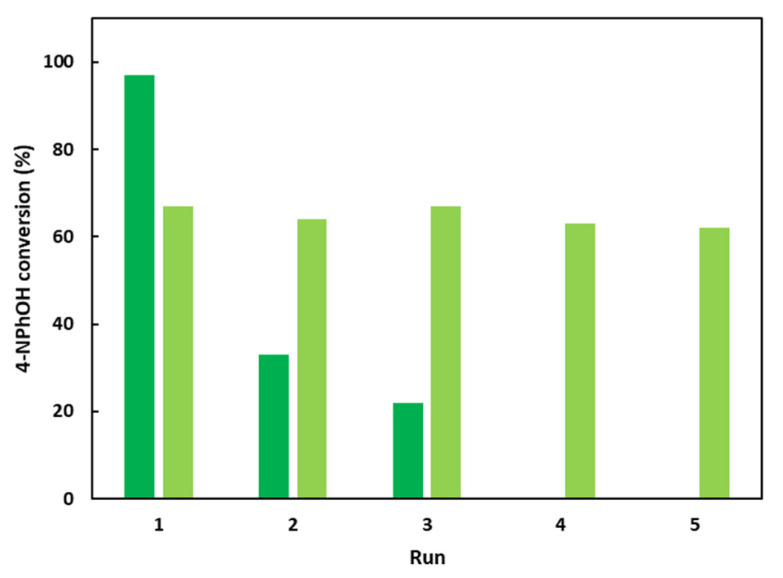
Recycling attempts for the 4-NPhOH reduction in NaBH_4_ aqueous solution in the presence of CoAl_CMβCD [0.06] or CoAl_CO_3_ with additional free CMβCD in the reaction medium. After a 30 min test, the suspension was centrifuged, and the recovered solid was washed and allowed to react for an additional test. Reaction conditions: ***dark green bars***: 4-NPhOH (2 µmol), NaBH_4_ (0.2 mmol), H_2_O (30 mL), CoAl_CMβCD [0.06] (initial weight = 20 mg); ***light green bars***: 4-NPhOH (2 µmol), NaBH_4_ (0.2 mmol), H_2_O (30 mL), CoAl_CO_3_ (initial weight = 15 mg), CMβCD (5 mg).

The hybrid LDH-based material has also been evaluated in terms of recycling (Figure 7). After 30 min, the solid (CoAl_CMβCD [0.06]) was recovered by centrifugation, washed several times with water, and finally dried at 60 °C under air for 24 h. A new 4-NPhOH solution in NaBH_4_ was prepared and the solid CoAl_CMβCD [0.06] was introduced.

The 4-NPhOH conversion after 30 min of reaction drastically decreased for the second run (33% vs. 97% for the first run) and this activity decrease continued for the third run (22%). This loss of activity could be explained by the gradual CMβCD loss with the experimental runs observed through TGA (ESI-Figure S4). Moreover, a new reaction was performed for 4 h and the supernatant was separated from the solid and was mixed with a new 4-NPhOH solution containing fresh sodium borohydride solution. A slower but noticeable 4-NPhOH conversion was observed, confirming a cobalt leaching which occurred during the run. Interestingly, according to XRD diffractograms of the recovered solid after several runs, the LDH phase was conserved (ESI-Figure S5). Interestingly, the CoAl-CO_3_ could be reused five times without activity loss by renewing for each run the 4-NPhOH solution in the presence of sodium borohydride and CMβCD.

## 3. Discussion

All these results clearly showed that CMβCD had a beneficial effect towards the 4-NPhOH reduction in an aqueous medium, either in the hybrid form or in the physical mixture form. The hybrid materials (cobalt-based LDH or cobalt-based hydroxide) showed higher activities in comparison with the cobalt-based materials without CMβCD. The apparent rate constants *k_app_* of CoAl_CMβCD [0.06], CoAl_CMβCD [0.03], PMCoAl_CO_3_/CMβCD [0.06] could be evaluated and were estimated to be 0.121, 0.116 and 0.042 min^−1^, respectively. Interestingly, these last values are higher than those obtained by Nunes et al. for the reduction of nitroaromatics using chloride-intercalated Co LDH (0.078, 0.064 and 0.050 min^−1^) [32] and those obtained by Meijboom et al. using mesoporous Co_3_O_4_ [16] or by Chen using Co_3_O_4_ without solid pretreatment [17] (Table 2). Concerning the experiments mixing the control materials with cyclodextrins, an enhanced activity was only observed for the LDH-based material and solely with CMβCD. Different hypotheses could be formulated to explain these results: (i) the 4-NPhOH adsorption on the layered material, (ii) the interactions between cobalt and CMβCD.

We have first examined the adsorption of 4-NPhOH in different LDH-based materials (CoAl_CO_3_, CoAl_CMβCD [0.06] and PMCoAl_CO_3_/CMβCD) by preparing a sodium borohydride solution of 4-NPhOH in the same concentration as that of the reduction experiment (0.2 mmol in 30 mL), monitoring by UV-Vis spectrophotometry (ESI Figure S6). In the presence of CoAl_CO_3_, less than 0.3% of 4-nitrophenolate was adsorbed on this material. The low adsorption capacity at pH = 9.2 has already been seen in literature [47] and could be explained by the occurrence of higher repulsive forces between the o-nitrophenolate anion (pKa = 7.23) and the negatively charged surface of the LDH-based adsorbent. In the case of PMCoAl_CO_3_/CMβCD, the presence of CMβCD improved the 4-nitrophenolate adsorption (1.6%). This small enhancement could be due to the possible inclusion complex between 4-NPhOH and CMβCD [34]. The best adsorption enhancement was obtained with the hybrid material where 9% of 4-NP was adsorbed.

**Figure 8:**
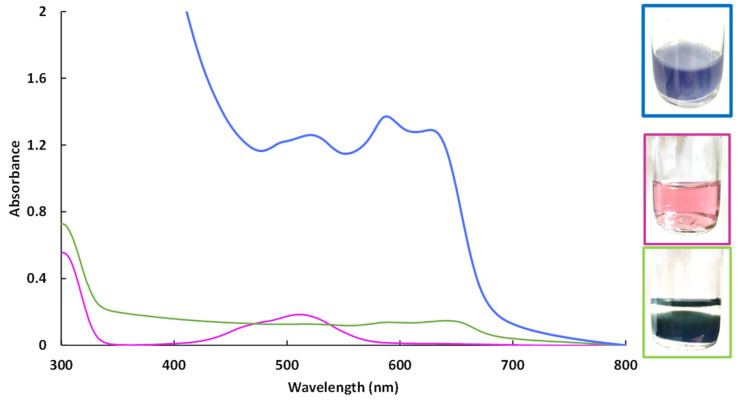
UV-Visible spectra of aqueous cobalt(II) solutions at pH = 10.5. Cobalt(II) nitrate solution at pH 6 (purple curve), cobalt(II) nitrate solution at pH 10.5 (green curve) and cobalt(II) nitrate solution at pH 10.5 with CMβCD (blue curve).

The better activities of the hybrid materials could be explained by different reducibility behaviors. For instance, CoAl_CMβCD and CoAl_CO_3_ LDH materials had different colors (purple and light brown respectively). These two colors indicated that different cobalt species have been obtained during the LDH synthesis. The stability of Co (II) has been studied by preparing a cobalt nitrate solution at the pH fixed during the coprecipitation to get the LDH, in presence or not of CMβCD (Figure 8).

Cobalt(II) ions, which precipitate in an alkaline solution (pH = 10.5), can be stabilized by CMβCD at the same pH value. According the UV-Vis spectrum of Co(II) in the presence of CMβCD at pH = 10.5, different cobalt species are present. During the precipitation step for getting the metal hydroxides (with or without Al(III)), a part of the cobalt (II) ions were stabilized by CMβCD, which could explain the color differences. During the 4-NPhOH reduction, these CMβCD-stabilized cobalt(II) ions were more sensitive towards the reducing agent, leading to the in-situ formation of cobalt nanoparticles. This hypothesis can be justified by the recycling study, where cobalt leaching had occurred (ESI Figures S7 and S9). However, the agreement could not be made if the activity is mainly due to nanoparticles dispersed in solution or immobilized onto the support. In contrast, without CMβCD incorporated in the material, the cobalt species were more stable towards the borohydride, especially the cobalt hydroxide Co(OH)_2_, which exhibited no activity for 4-NPhOH reduction. The poor activity of the Co hydroxide/oxide forms has already been observed in a previous study, using Co_3_O_4_ as a cobalt source for the reduction of 4-NPhOH [17]. According to this study, the authors also evidenced that the activity of bulk Co_3_O_4_ could be significantly enhanced by performing a pretreatment on the material in the presence of NaBH_4_. The performance in the reduction for the 4-NPhOH was shown to be strongly dependent on the duration of this pretreatment: the longer the duration, the higher the activity. The authors justified these improved activities through the creation of oxygen vacancies in the cobalt oxide matrix, facilitating the cobalt species reduction. According to the UV-vis spectrophotometry analyses, we could make the hypothesis that CMβCD strongly interacts with the cobalt (II) centers and modify the redox behavior and reducibility of cobalt, maybe yielding up to complete reduction. Our group has previously established that the controlled addition of β-cyclodextrin could have a significant beneficial impact on the dispersion and reducibility of supported cobalt species. A last test was performed to confirm the importance of the cyclodextrin backbone. The 4-NPhOH reduction was performed in the presence of 3.5 molar equivalents of potassium cellobionate (the chemical equivalent of CMβCD) [48,49] but no activity enhancement was observed. The cyclodextrin backbone with strong complexing groups such as carboxylate groups seems to be essential.

**Figure 9:**
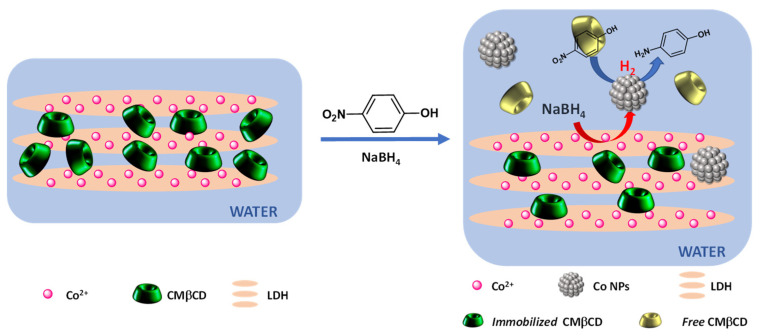
Proposed mechanism for the enhanced activity of CoAl_CMβCD.

It can be concluded that the efficiency of the hybrid cobalt-based materials was due to the strong electrostatic interactions between Co(II) and the carboxylate groups. These interactions influenced the cobalt reducibility and led, in the presence of NaBH_4_, to the formation of active cobalt nanoparticles (Figure 9). This efficiency can also be partly explained by the presence of the cyclodextrin cavity, which presents the ability to form inclusion complexes with 4-NPhOH [50].

## 4. Materials and Methods

### 4.1. Chemicals

Sodium borohydride, sodium carbonate, aluminum (III) nitrate nonahydrate were purchased from Aldrich. Cobalt (II) nitrate hexahydrate was purchased from Strem Chemicals Inc. β-cyclodextrin (βCD) was kindly supplied by Roquette Freres (Lestrem, France). Randomly methylated β-cyclodextrin (RaMeβCD) with an average degree of substitution of 1.8 methyl groups per glucopyranose unit (MW 1310 g·mol^−1^) was a gift from Wacker Chemie GmbH (Lyon).

### 4.2. Synthesis of Materials

#### 4.2.1. Synthesis of Carboxymethyl β-Cyclodextrin

10 g of β-cyclodextrin were dissolved in a 1.25 M sodium hydroxide solution (74 mL). 54 mL of a chloroacetic acid solution (4.4 g) were added in the previous solution. The resulting solution was stirred at 50 °C for 48 h. After cooling, the solution was neutralized with a hydrochloric acid solution and the modified cyclodextrin was precipitated in an ethanol-isopropanol mixture (1.2 L), centrifugated, and dried under vacuum for 72 h. The substitution degree of CMβCD was determined by ^1^H NMR spectroscopy and was estimated to be 3.2 carboxylate groups per cyclodextrin molecule (MW 1391 g·mol^−1^) (ESI-Figure S1).

#### 4.2.2. Synthesis of Cobalt-Based LDH

- CoAl_CO_3_ LDH

20.95 g of Co(NO_3_)_2_·6H_2_O and 10.11 g of Al(NO_3_)_3_·9H_2_O were dissolved in 200 mL of deionized water and the resulting metallic cation solution was poured in an addition funnel (solution A). 9.54 g of Na_2_CO_3_ were dissolved in 300 mL of deionized water and put in a beaker (solution B). Solution A was dropwise added into solution B for 10 min. The pH of the mixture was set at a value of 10.5 by using a 2 mol·L^−1^ sodium hydroxide solution. When the addition was complete, the resulting suspension was stirred at 60 °C for 24 h. The precipitate was filtrated, washed with hot deionized water, and dried for 64 h at 60 °C. 9.87 g of a beige solid (yield = 99%) was obtained and crushed in a mortar. The resulting material is denoted as CoAl_CO_3_.

- CoAl_CMβCD[X] LDH

8.38 g of Co(NO_3_)_2_·6H_2_O and 4.04 g of Al(NO_3_)_3_·9H_2_O were dissolved in 80 mL of deionized water and the resulting metallic cation solution was poured in an addition funnel (solution A). 9.90 g or 4.95 g of CMβCD were dissolved in 120 mL of deionized water and put in a beaker (solution B), (corresponding respectively to 0.06 and 0.03 mol·L^−1^ CMβCD molar concentrations). Solution A was dropwise poured into solution B for 10 min. The pH of the mixture was kept at a value of 10.5 by using a 2 mol·L^−1^ sodium hydroxide solution. When the addition was complete, the resulting suspension was stirred 24 h at 60 °C, filtrated, and washed with deionized water until filtrate pH corresponded to the water pH. The solid was dried for 64 h at 60 °C. A pink-purple solid was obtained (10.24 g with [CMβCD] = 0.06 mol·L^−1^ and 10.52 g with [CMβCD] = 0.03 mol·L^−1^) and crushed in a mortar. The resulting hybrid materials are denoted as CoAl_CMβCD[X] with X, the initial CMβCD concentration.

### 4.3. Characterization Methods

#### 4.3.1. Nuclear Magnetic Resonance Spectroscopy

NMR spectra were recorded at 298 K on a Bruker Avance III HD 300 NanoBay spectrometer equipped with a 5 mm broadband probe BBFO with Z-gradients, operating at 7.05 T field strength at 300 MHz for ^1^H nucleus. D_2_O (99.92% isotopic purity) was purchased from Euriso-Top.

#### 4.3.2. Powder *X*-ray Diffraction

Powder X-ray diffraction data were collected on a Siemens D5000 X-ray diffractometer (Bruker AXS, Palaiseau, France) in a Bragg-Brentano configuration with a Cu Ka radiation source. Scans were run over the angular domains 5°< 2θ < 80° with a step size of 0.02° and a counting time of 2 s/step. Crystalline phases were identified by comparing the experimental diffraction patterns to Joint Committee on Powder Diffraction Standards (JCPDS) files. The treatment of the diffractograms was performed using the FullProf software and its graphical interface WinPlot.

#### 4.3.3. Thermogravimetric Analysis (TGA) Coupled with Differential Scanning Calorimetry (DSC)

Thermogravimetric Analysis coupled with Differential Scanning Calorimetry was measured using a simultaneous TGA/DSC3+ instrument from Mettler Toledo coupled with a computer having STARe software. Approximately 10 mg of the sample was heated in an open 70 μL alumina crucible from 40 to 800 °C (10 °C·min^−1^) under air at a flow rate of 50 mL min^−1^.

#### 4.3.4. Fourier Transform Infrared (FTIR) Spectroscopy

Fourier transform infrared (FTIR) experiments were performed using a Spectrum Two Perkin-Elmer FTIR spectrometer equipped with a single-reflection diamond module (ATR) and a deuterated triglycine sulfate detector. The FTIR spectra were recorded in the wave number range of 400–4000 cm^−1^.

#### 4.3.5. Scanning Electron Microscopy (SEM)

Measurements were performed using a Hitachi SU3800 (Hitachi, Tokyo, Japan) operating at 15 keV accelerating voltage and in the low vacuum mode (30 Pa), equipped with a backscatter electron (BSE) detector and energy dispersive X-ray spectrometer (EDS). In order to determine the elemental percentage for each sample, four arbitrarily areas of the images were chosen and the average percentage was calculated.

#### 4.3.6. UV-Vis Spectrophotometry

UV-Vis measurements were carried out with a Shimadzu 2600 UV-vis spectrophotometer and the spectra were evaluated using UV Probe 2.62 software from Shimadzu company.

### 4.4. 4-Nitrophenol Reduction Procedure in Aqueous Medium

2 mL of a 2mM 4-NPhOH aqueous solution were added in a 50 mL-round bottom flask containing 8 mL of water. 20 mL of a 20 mM sodium borohydride aqueous solution was quickly added in the previous solution, instantly followed by the addition of 20 mg of CoAl-based LDH. The resulting mixture was stirred with a magnetic barer for 30 min. Kinetic monitoring was performed by taking aliquots of the reaction medium and analyzing them immediately by UV-Vis spectrophotometry.

## 5. Conclusions

Cobalt-based LDH materials in the presence of carboxymethyl-β-cyclodextrin were synthesized through a one-pot coprecipitation approach. The physicochemical characterization of our materials carried out by means of XRD, TGA, FTIR, and EDX-SEM gave valuable information about the crystalline structure, functional groups, and chemical composition, even though other techniques, such as the solid-state NMR spectroscopy, could provide a more detailed description about the local environment [51]. These hybrid materials were tested in the reduction of 4-nitrophenol in an aqueous medium. The hybrid samples have shown higher activities in comparison with the material without cyclodextrin (CoAl_CO_3_) and with the physical mixtures of this latter with different cyclodextrins. Several control experiments were realized and emphasized the crucial role of the CMβCD through complemental strong electrostatic interactions which facilities the cobalt reducibility but also the inclusion complex with 4-NPhOH thanks to the cyclodextrin cavity.

## Figures and Tables

**Table 1:** EDS Analyses of cobalt-based LDH materials.

Sample	wt % Co (*)	wt % Al (*)	wt % C (*)	wt % O (*)	wt % Na (*)	wt %Si (*)	wt % Cl (*)
CoAl_CO_3_	46.3 ± 0.9 (43.6)	5.6 ± 0.1 (6.6)	3.8 ± 0.2 (1.5)	43.5 ± 1.1 (45.3)		0.4 ± 0.01	
CoAl_CMβCD [0.03]	39.3 ± 0.6 (31.5)	5.2 ± 0.2 (4.8)	13.5 ± 0.4 (13.2)	40.3 ± 0.6 (49.9)	1.0 ± 0.1 (0.6)		0.7 ± 0.02
CoAl_CMβCD [0.06]	38.6 ± 1.5 (30.3)	4.8 ± 0.2 (4.6)	13.7 ± 0.9 (14.6)	41.5 ± 1.2 (49.8)	1.0 ± 0.1 (0.7)		0.5 ± 0.05

*: theoretical wt % value.

**Table 2:** Apparent rate constants of different cobalt-based materials for the reduction of 4-NPhOH by NaBH_4_.

Material	Molar Ratio NaBH_4_/4NPhOH	k_app_ (min^−1^)	Reference
Meso Co_3_O_4_	250	0.018	[16]
Co_3_O_4_	100	0	[17]
Reduced Co_3_O_4_	100	1.49	[17]
CoAl_Cl	100	0.064 *	[32]
CoAl_CMβCD	100	0.116–0.121	*Our work*

* Average apparent rate constants.

## Data Availability

Data are contained within the article and Supplementary Materials.

## Supplementary Materials

The supporting information can be downloaded at: https://www.mdpi.com/article/10.3390/ijms25126390/s1.
