# Supplementary material for: Carboxymethyl β-Cyclodextrin Assistance for the 4-Nitrophenol Reduction Using Cobalt-Based Layered Double Hydroxides
Source: Int J Mol Sci. 2024 Jun 9;25(12):6390. doi: 10.3390/ijms25126390 (PMC11203548; doi:10.3390/ijms25126390)
Supplement: Supplementary file 1 [file ijms-25-06390-s001.zip › ijms-2979833-supplementary.pdf]

Supporting Information

Carboxymethyl β-Cyclodextrin Assistance for the 4-Nitrophenol Reduction Using Cobalt-Based Layered Double Hydroxides

Alexia Demeester [1], Fatima Douma [1], Renaud Cousin [2], Stéphane Siffert [2], Gwladys Pourceau [3], Anne Wadouachi [3], Anne Ponchel [1], Eric Monflier [1] and Sébastien Noël [1,]*

1 Unité de Catalyse et Chimie du Solide (UCCS), UMR 8181, Université de Lille, CNRS, Centrale Lille, Université d'Artois, rue Jean Souvraz, SP 18, 62300 Lens, France

2 Unité de Chimie Environnementale et Interactions sur le Vivant (UCEIV, UR 4492), Université du Littoral Côte d'Opale, 59140 Dunkerque, France.

3 Laboratoire de Glycochimie et des Agroressources d'Amiens (LG2A) UR 7378, Institut de Chimie de Picardie, Université de Picardie Jules Verne, 33 rue St Leu, 80039 Amiens, France

* Corresponding authors
E-mail : sebastien.noel@univ-artois.fr
Phone: +33 (0)3 21 79 17 49
Fax: +33 (0)3 21 79 17 55

Table of Contents

I) Determination of the CMβCD amount inside the LDHs — **p. S2**

II) Synthesis of cobalt-based hydroxides — **p. S3**

Figure S1. 1H NMR spectrum of CMβCD (D2O, 25 °C, 300 MHz) — **p S4**

Figure S2. Scanning electron micrographs and corresponding atomic composition of CoAl-based LDH materials — **p S5**

Figure S3. UV-Vis spectrum of an equimolar mixture of 4-nitrophenol and 4-aminophenol in a sodium borohydride aqueous solution — **p. S6**

Figure S4. Thermogramms of CoAl_CMβCD[0.06] — **p S7**

Figure S5. Diffractogramms of CoAl_CMβCD[0.06] in recycling study — **p S8**

Figure S6. 4-NPhO$^-$ adsorption on cobalt-based materials — **p. S9**

Figure S7. CoAl_CMβCD[0.06] recycling for the 4-NPhOH reduction monitored by UV-Vis spectrophotometry — **p S10**

I) Determination of the CMβCD amount inside LDHs

The amount of CMβCD can be estimated thanks to the corresponding TGA profile but also with different hypothesis that have to be considered.

Hypothesis 1: the anionic cyclodextrin (CMβCD with a substitution degree of 3.2 is linked twice with the 2 cationic layers (like the carbonate anion). There is 1.2 carboxylate group left which is finally linked with 1.2 sodium cation.

The molecular weight can be calculated as follows:

M (CMβCD in LDH) = (βCD) – 3.2*M(H) + 3.2 M(CH_2COO) + 1.2*M(Na)

M (CMβCD in LDH) = 1135 -3.2*1 +3.2*(14+12+32)+1.2*23.

M = 1345 g/mol

Hypothesis 2: the hybrid LDH can be composed with CMβCD but also carbonate ions.

Taking into account the general molecular formula $[M(II)_a\, M'(III)_b\, (OH)_{2a+2b}]\, (A^{n-}_{b/n}).4\, H_2O$ and the b value of 2 (hypothesis 1) : $[Co_6Al_2(OH)_{16}](CO_3^{2-}$ and/or CMβCD). 4 H_2O

M (hybrid LDH) = 6*M(Co) + 2*M(Al) + 16*M(OH)+X*M(CMβCD in LDH) +(1-X)*M(CO_3) with X the molar ratio of intercalated CMβCD

M (hybrid LDH) = 6*59 + 2*27 + 16*17 + X*1345 +(1-X)*60 + 4*18

M (hybrid LDH) = 812 + 1285X

Hypothesis 3: the calcinated product obtained during the TGA analysis under air is the same for control and hybrid LDHs, i.e. **$Co_6Al_2O_{11}$**, so **584 g/mol**.

- For the hybrid material CoAl_CMβCD[0.06], the final weight is 51%, so the initial weight is Mi = $\frac{584}{0.51}$= **1168 g/mol.** With the previous equation, 812 + 1285 X = 1168, so **X = 0.28**
- For the hybrid material CoAl_CMβCD[0.03], the final weight is 52%, so the initial weight is Mi = $\frac{584}{0.52}$= **1123 g/mol.** With the previous equation, 812 + 1285 X = 1123, so **X = 0.24**

CONCLUSION: the estimated amount of CMβCD

in CoAl_CMβCD[0.06] is 28%

in CoAl_CMβCD[0.03] is 24%

II) Synthesis of cobalt-based hydroxides

Without CMβCD

3.13 g of $Co(NO_3)_2.6H_2O$ were dissolved in 20 mL of deionized water and the resulting metallic cation solution was poured in an addition funnel and dropwise poured into 30 mL of an alkaline solution during 10 minutes. The pH of the mixture was kept at a value of 10,5 by using a 2 $mol.L^{-1}$ sodium hydroxide solution. When the addition was complete, the resulting suspension was stirred 24h at 60°C, filtrated, washed with deionized water until filtrate pH corresponded to the water pH. The solid was dried during 64h at 60°C. A dark grey solid (0.97 g) was obtained and crushed in a mortar.

With CMβCD at an initial concentration of 0.06M

3.13 g of $Co(NO_3)_2.6H_2O$ were dissolved in 20 mL of deionized water and the resulting metallic cation solution was poured in an addition funnel (solution A). 1.30 g of CMbCD, dissolved in 30 mL of an alkaline solution, were put in a becher with a fixed pH of 10,5 (solution B). Solution A was dropwise poured into solution B during 10 minutes. The pH of the mixture was kept at a value of 10,5 by using a 2 $mol.L^{-1}$ sodium hydroxide solution. When the addition was complete, the resulting suspension was stirred 24h at 60°C, filtrated, washed with deionized water until filtrate pH corresponded to the water pH. The solid was dried during 64h at 60°C. An olive green solid (1,16 g) was obtained and crushed in a mortar.

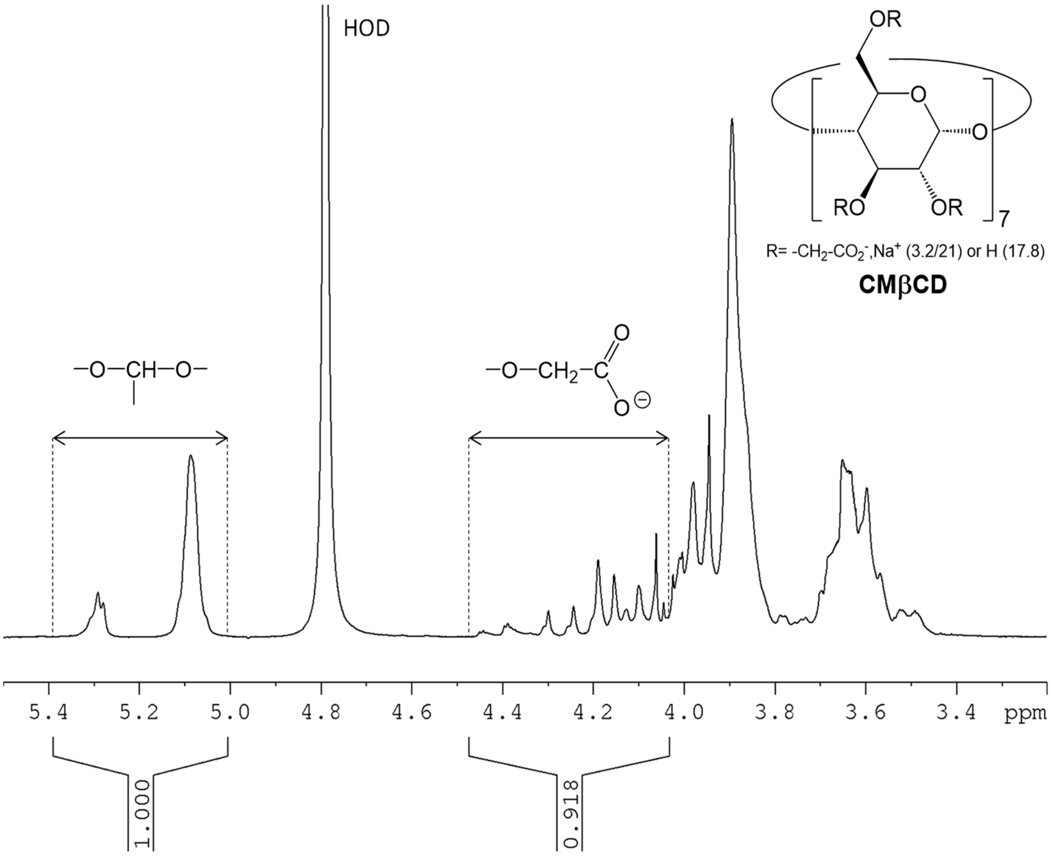

Figure S1. **^{1}H NMR spectrum of CMβCD (D_2O, 25 °C, 300 MHz)**

Using the ^{1}H NMR spectrum of CMβCD it is possible to determine the average degree of substitution (DS) of this cyclodextrin. In the case where the integration of the signals of the H_1 protons of the CD is calibrated to 1 (1 H_1 proton per glycosidic unit), the integration of the CH_2 signals of the carboxymethyl groups gives the average number of protons present on this modified cyclodextrin. The DS was found by dividing this integration by 2 and multiplying by 7 (the number of glycosidic units). Using this calculation, we obtain an average degree of substitution equal to 3.2.

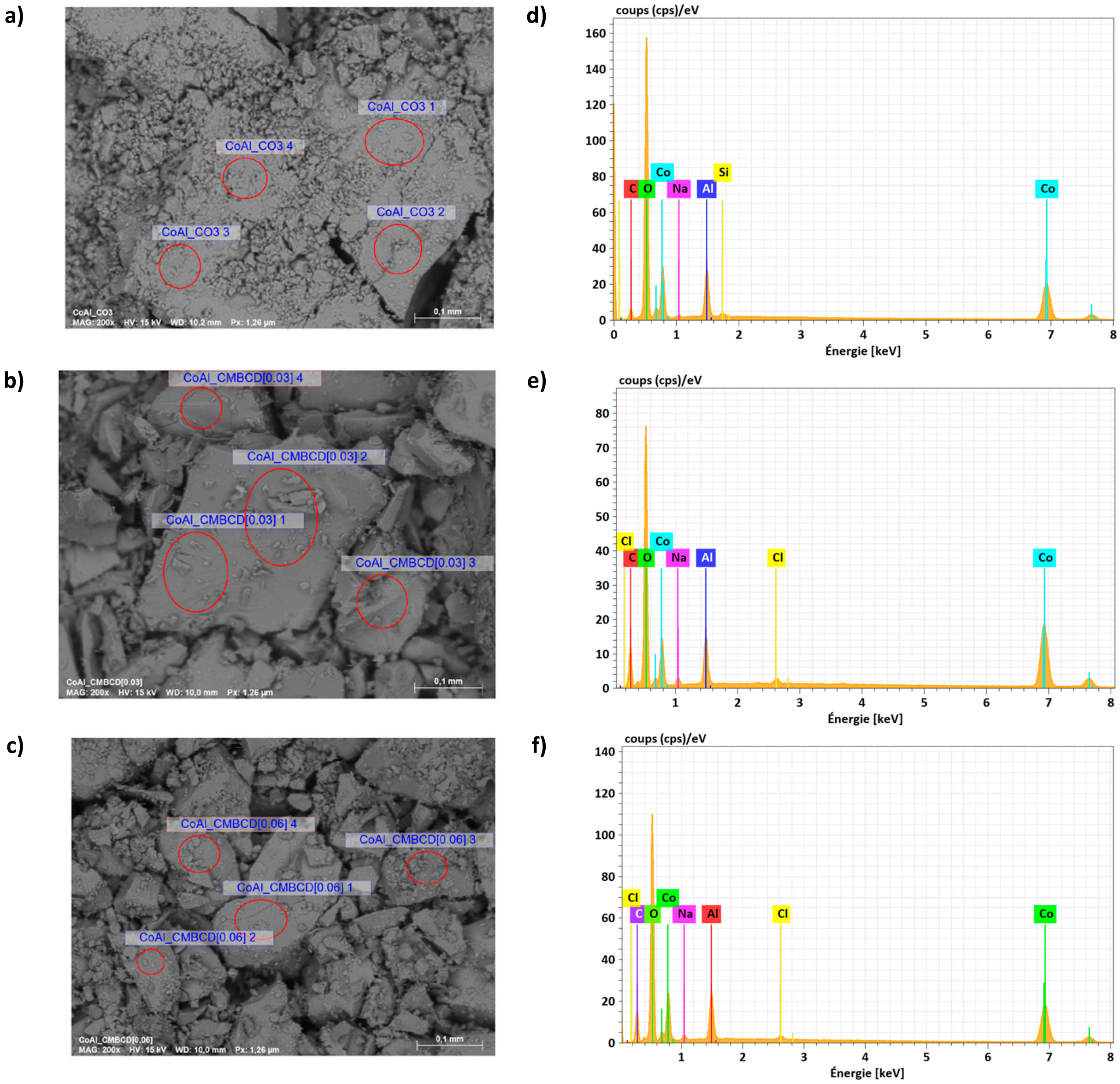

Figure S2. Scanning electron micrographs and the corresponding average elemental analysis of the four arbitrary domains through EDX of CoAl_CO_3 (**a**,**d**), CoAl_CMβCD[0.03] (**b**,**e**) and CoAl_CMβCD[0.06] (**c**,**f**).

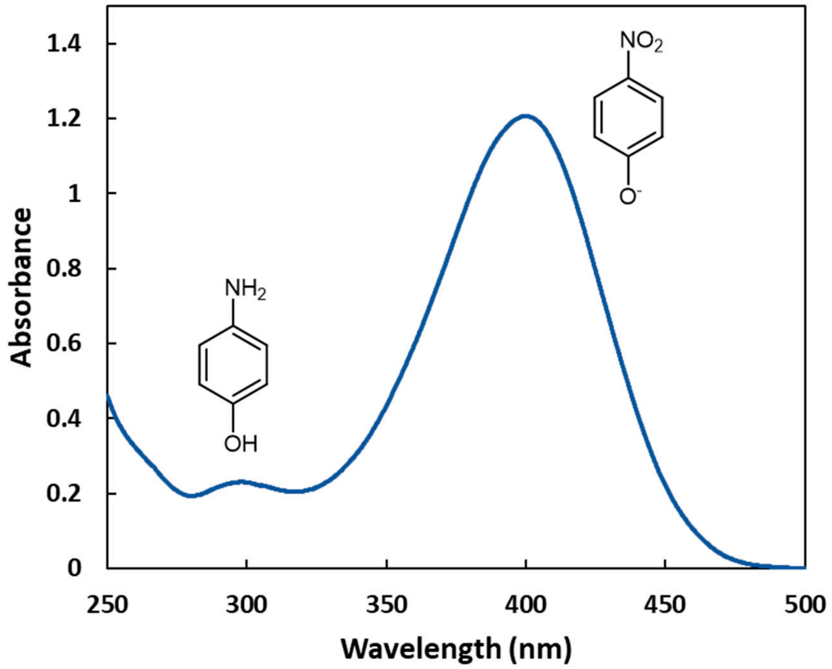

Figure S3. UV-Vis spectrum of an equimolar mixture of 4-nitrophenol and 4-aminophenol in a sodium borohydride aqueous solution

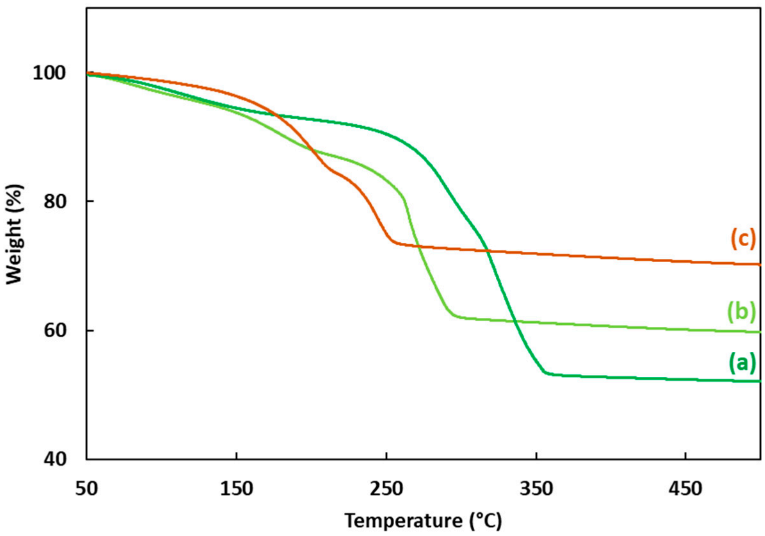

Figure S4. Thermogramms of CoAl_CMβCD[0.06] (fresh (a); after the 3rd run (b), CoAl_CO_3 (c))

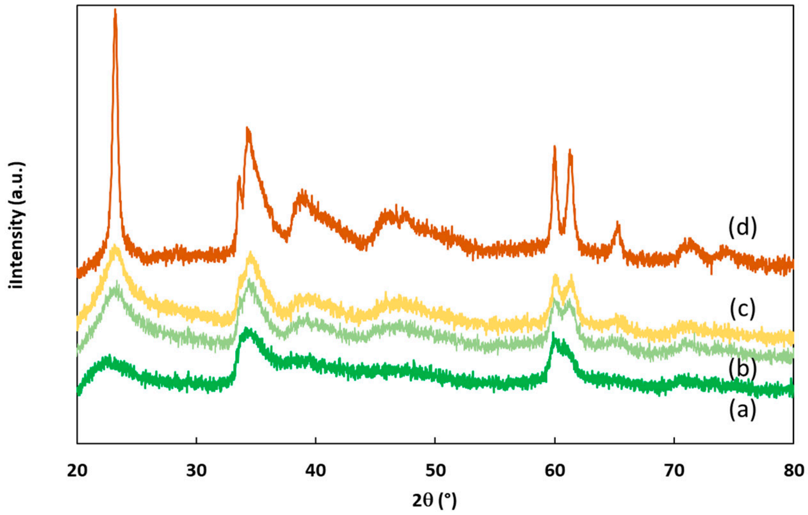

Figure S5. Diffractograms of CoAl_CMβCD[0.06] during recycling study (fresh (a); after the 1st run (b), after the 3rd run (c), and CoAl_CO_3 diffractogram (d))

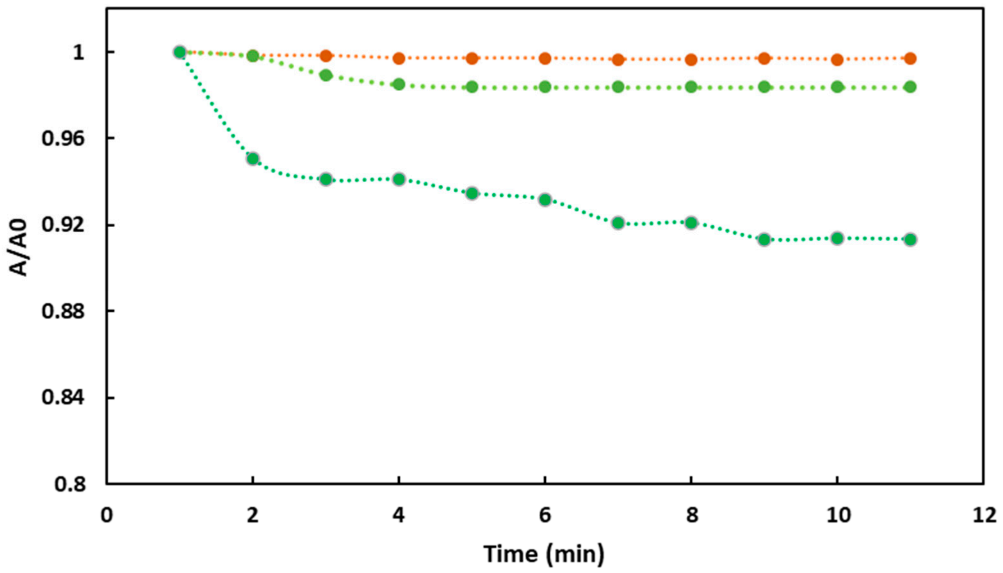

Figure S6. 4-NPhO$^-$ adsorption on cobalt-based materials (CoAl_CO$_3$ (brown line); CoAl_CO$_3$ + CMβCD (light green line); CoAl_CMβCD[0.06] (dark green line))

III) Material Recycling procedure

After 30 minutes of reaction (RUN 1), the suspension is centrifugated and the supernatant was removed. This supernatant was mixed with a fresh 4-NPhOH solution containing sodium borohydride (RUN 2) The kinetic of the reaction was monitored by UV-Vis spectrophotometry.

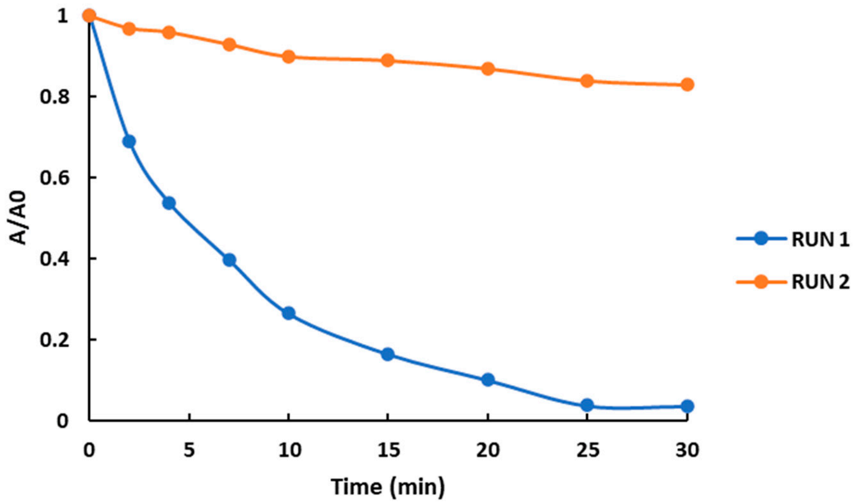

Figure S7. CoAl_CMβCD[0.06] recycling for the 4-NPhOH reduction monitored by UV-Vis spectrophotometry
